# Myocardial perfusion imaging with ^99 m^Tc - tetrofosmin SPECT in breast cancer patients that received postoperative radiotherapy: a case-control study

**DOI:** 10.1186/1748-717X-6-151

**Published:** 2011-11-08

**Authors:** Chrissa Sioka, Thomas Exarchopoulos, Ifigenia Tasiou, Eftychia Tzima, Nikolaos Fotou, Antonio Capizzello, Vasilios Ragos, Periklis Tsekeris, Andreas Fotopoulos

**Affiliations:** 1 Department of Nuclear Medicine, University Hospital of Ioannina, Greece; 2 Department of Radiation Therapy, University Hospital of Ioannina, Greece; 3 Department of Medical Physics, University Hospital of Ioannina, Greece; 4 Department of Maxillofacial Surgery, University Hospital of Ioannina, Greece

**Keywords:** Myocardium, SPECT, Breast cancer, Radiotherapy

## Abstract

**Purpose:**

To evaluate the cardiac toxicity of radiotherapy (RT) in breast cancer (BC) patients employing myocardial perfusion imaging (MPI) with Tc-99 m Tetrofosmin - single photon emission computer tomography (T-SPECT).

**Materials and methods:**

We studied 46 BC female patients (28 patients with left and 18 patients with right BC) treated with postoperative RT compared to a control group of 85 age-matched females. The median time of RT to SPECT was 40 months (6-263).

**Results:**

Abnormalities in the summed stress score (SSS) were found in 54% of left BC patients, 44.4% of right BC patients, and 32.9% of controls. In left BC patients there were significantly more SSS abnormalities compared to controls (4.0 ± 3.5 vs 2.6 ± 2.0, p = 0.05) and possible trend of increased abnormalities of right BC patients (3.7 ± 3.0 vs 2.6 ± 2.0, p = 0.14). Multiple regression analysis showed more abnormalities in the MPI of left BC patients compared to controls (SSS, p = 0.0001); Marginal toxicity was also noted in right BC patients (SSS, p = 0.045). No additional toxicity was found in patients that received adjuvant cardiotoxic chemotherapy. All T-SPECT abnormalities were clinically silent.

**Conclusion:**

The study suggests that radiation therapy to BC patients result in MPI abnormalities but without apparent clinical consequences.

## Introduction

Breast cancer (BC) is the most common cancer in women [1]. Postsurgical radiation therapy offers substantial benefits in appropriately selected patients with BC [2-4]. Because radiation techniques have improved over time, the risk of death from ischemic heart disease associated with radiation for BC has substantially decreased over time [5]. The delivery of radiation is based on the anatomic volumes from CT simulation scans thus all tissue at risk must be meticulously delineated to allow the dose of radiation to be sculpted to the target structures (ie breast, chest wall ± regional nodes) while minimizing dose to the normal structures (ie heart and lung). Using CT simulators, modern-day linear accelerators, computerized treatment planning modalities, and on-board imaging techniques, the therapeutic ratio for radiation therapy has markedly improved, whereas the potential for side effects has diminished significantly. However, radiation techniques that include both breast and regional lymph nodes, result in a higher probability of heart complications compared with tangential irradiation of the breast only [6]. In any event, radiotherapy for left sided BC may represent an independent risk factor in the long-term development of ischemic heart disease [7], and patients who receive high irradiation dose-volumes may exhibit increased mortality due to radiation-induced microvascular damage to the heart [8].

Myocardial perfusion abnormalities both at rest and after stress are reliable predictors of subsequent cardiac events in patients with ischemic heart disease [9,10]. In order to better understand the cardiac toxicity of modern radiation therapy, we compared myocardial perfusion imaging (MPI) defects in BC patients who received radiation therapy to MPI defects in age-matched women with no prior radiotherapy.

## Patients and methods

### Patients

Forty six female patients with primary BC that had been treated with postoperative adjuvant radiation therapy (RT) from 1998 to 2010 were subjected after stress and at rest to MPI, employing Tc-99 m Tetrofosmin - single photon emission computer tomography (T-SPECT). All patients at the time of the test were in remission and none had any history or symptoms of coronary artery (CAD) or other heart disease. Among them, 28 patients had prior RT to the left and 18 patients to the right-breast area. Furthermore, 85 age-matched control females with no medical history of cancer or RT or known CAD were subjected to diagnostic MPI with T-SPECT. These control individuals were females that were referred for T-SPECT because of non-specific complaints of various type chest wall pains, palpitations, or shortness of breath, and negative clinical cardiology examination and normal ECG. All participants were interviewed in person using a structured questionnaire. Major cardiac risk factors including age, smoking, hypertension, diabetes, dyslipidemia and family history of cardiovascular disease were recorded. Arterial hypertension was documented when systolic blood pressure (BP) was ≥ 140 mmHg or diastolic BP ≥ 90 mmHg or when individuals were receiving anti-hypertensive drugs for previously established hypertension. Smoking, active or ceased within the last 3 months, was considered as current. Diabetes mellitus was considered as present if fasting glucose was > 126 mg/dl or the individual was treated with antidiabetic medication. Dyslipidemia was defined as fasting cholesterol > 220 mg/dl or the individual was on current treatment with specific agents. Information concerning their BC history (*i.e.*, side of radiation therapy, age during the therapy, previously use of chemotherapy etc) was collected. The protocol was approved by the Hospital's Clinical Research Committee and all studied individuals gave informed consent to the examination.

### Radiation therapy

The treatment planning was performed using a 3D treatment planning system (Pinnacle version 7.4). Treatment plans were normalized at the International Commission on Radiation Units and Measurements (ICRU) reference point of the breast Planning Target Volume (PTV). Every patient was immobilized in a supine position and placed on an inclined breast board in an optimized treatment position. Patients were simulated in a large-bore CT simulator with 8 mm slice separation. Missing tissue compensation filters (wedges) were used when it was necessary. The heart was contoured using the soft-tissue shadow at one slice below the inferior cut of the right pulmonary artery crossing the midline. After delineation of the target and organs at risk, CT data were transferred to the treatment planning system.

For the tangential field technique, two 2D-radiographic based parameters related to volumetric heart dose were measured: the Maximum Heart Distance (MHD), i.e. the maximum distance of the heart contour to the medial field end, as can be seen on a beam's eye view of the mediolateral tangential field and the Maximum Heart Length (MHL), i.e. the maximum length of the heart in the tangential fields. MHD and MHL were used to estimate the volume of heart receiving high doses using tangential fields. Three dimensional dose distributions and DVHs of PTV and heart were analyzed.

Τhe irradiation was delivered with two tangential fields (a medial and a lateral one) with a linear accelerator (6 MV). According to the type of operation (breast conserving surgery or mastectomy) the whole breast with or without the chest wall was included in the treatment fields. The irradiation was delivered in 30 fractions over a 6-week period. The total prescribed dose was 60 Gy (50 Gy to the PTV and 10 Gy as a boost to the tumor bed). The daily dose was 2 Gy. According to the pathology report, radiotherapy was given to the axillary and internal mammary lymph nodes. These areas if treated were included in the tangential fields. Approximately 2 cm of lung between the medial border of the field and the chest wall, where is considered appropriate to provide an adequate field margin for chest wall irradiation, was included in our fields. The breast PTV was outlined on each CT-slice and defined according to ICRU-62 guidelines. The dorsal edges of both beams were made coplanar to decrease the amount of lung tissue irradiated.

Τhe mean breast volume was 667.1 cm^3 ^(range 503-808 cm^3) ^and the mean heart dose was on average 774.3 cGy (range 484.5-1095.5 cGy). Percentage volume (PV) of heart at different dose levels correlated fairly with MHD (cm) as the following relationships: PV20 = 5.4 MHD - 0.3 (R = 0.94); PV40 = 4.0 MHD - 0.6 (R = 0.89). The percentage volume of the heart at 20% and 40% of the total dose was about 4.7 times the MHD (cm) whereas the mean heart dose (7.743 Gy) approximated 4.8 times the mean MHD value (1.62 cm). The mean MHL value was 4.8 cm for the whole treatment and 8.3 cm for the 25 sessions (boost sessions not included). Parameters related to heart were only measured for left breast irradiation.

### SPECT MPI

All study individuals were subjected prior and after stress to T-SPECT using a 1-d imaging protocol. None of the patients or the control individuals had gated-SPECT. Stress protocol was either pharmacological (dipyridamole or dobutamine) or with dynamic exercise (Bruce protocol treadmill exercise test) according to local practice and clinical indication. Stress protocol and imaging was performed according to published guidelines [11]. An electrocardiograph was recorded at rest and continuously monitored during stress.

All participants were injected with 8 mCi Tc-99m-tetrofosmin bolus after stress and 20 mCi at rest, flushed with normal saline, and 30 - 40 minutes later the SPECT images were acquired. All studies were completed with a 90°-angled dual-head camera, using a low-energy, all-purpose collimator with 64 stops and 25 s per projection (30 projection/head) over a 180° arc. Acquisition was obtained with a matrix size of 64 × 64 and with a 15% symmetric window at 140 keV. The method of reconstruction consisted of filter back projection (filter butterworth, cut-off frequency 0.4, power 10). No attenuation correction was used.

Two nuclear medicine specialists, blinded to the participant's medical condition, visually evaluated the MPI using both a 19 segment [12] and the 17 segment [13] polar maps for the left ventricle. Both MPI polar maps had identical results. Each segment was scored on a scale of 0 to 5 according to the severity of the myocardial perfusion deficit. If there was no decreased myocardial activity it was scored 0, for mildly activity it was scored 1, for mild to moderate decreased activity as 2, for moderately to severely decreased activity as 3, for severely activity as 4 and with absent tracer activity as 5 points. Thus, the individual scores of perfusion defects within each 20 segments at rest and during stress were combined and provided the summed rest score (SRS) and summed stress score (SSS). According to the summed stress score the myocardial ischemia was graded as mild (4-8), moderate (9-13), and severe (> 13) [14]. Furthermore, the difference of the two scores (S-RSS) and myocardial anterior wall summed stress score (ASSS) were evaluated as well.

### Statistical analysis

The analyzed MPI scores of BC patients that were irradiated in the left side were compared to those that were irradiated in the right side. In addition, comparison was made between irradiated patients and control individuals. Separate comparisons were made between left and right BC patients with the control group of 85 individuals. The role of chemotherapy, time elapsed from RT, breast type operation and cardiovascular risk factors with MPI scores were also analyzed.

The results are shown as mean ± SD. The differences between the groups were tested for significance using the Student's t-test for independent samples. Stepwise multiple regression analysis was performed to examine variables independently associated with SSS, SRS and ASSS after controlling for cardiovascular risk factors. Left or right BC radiotherapy versus controls and left versus right BC radiotherapy were included in the model, assumed to be independent variables. P ≤ 0.05 was considered as statistically significant. Data were analyzed with the SPSS 13.0 for windows program package.

## Results

The physical characteristics of patients and control group that were matched for age are shown in Table 1. There were no statistical differences in the age of the BC patients of either subgroup (left or right BC) or control group at the time of the SPECT. The median time from BC irradiation to SPECT was 35 months (range, 6-142) for the patients with left BC and 41 months (range 6-263) for the group with right BC. Among the 46 BC patients, 26 had radical mastectomy (17 in left BC group and 9 in right BC group), and 20 patients had only tumor removal without mastectomy (11 patients in left and 9 patients in right breast). In 16 of the 29 patients who received adjuvant chemotherapy the drug regimen included a cardiotoxic agent (either epirubicin or adriamycin). Comparison of SPECT-MPI results in patients that either received adjuvant cardiotoxic chemotherapy or not showed no significant differences among the two groups (Table 2).

**Table 1:** Physical characteristics of patients with left and right breast cancer and control individuals

	Left Breast Cancer N = 28	Right Breast Cancer N = 18	All Breast Cancer patients N = 46	Control individuals N = 85
**Mean age (SPECT)**	59.93 ± 10.57 (41-79)	57.50 ± 9.34 (40-78)	58.97 ± 10.08 (40-79)	58.72 ± 9.72 (40-80)
**Body weight (kg)** **(SPECT)**	70.39 ± 10.66 (52-95)	72.94 ± 10.70 (53-95)	71.39 ± 10.63 (52-95)	71.72 ± 10.81 (40-105)
**Mean age (RT)**	56.46 ± 10.91 (41-78)	53.17 ± 8.53 (36-71)	55.17 ± 10.08 (36-78)	_
**Median time from RT to SPECT (months)**	35 (6-142)	41 (6-263)	40 (6-263)	_
**Type of breast surgery** **(M/O)**	M: 17/28 (60.7%) O: 11/28 (39.3%)	M: 9/18 (50.0%) O: 9/18 (50.0%)	M: 26/46 (56.5%) O: 20/46 (43.5%)	_
**Chemotherapy**	17/28 (60.7%)	12/18 (66.7%)	29/46 (63.0%)	_
**Cardiotoxic** **Chemotherapy**	11/28 (39.3%)	5/18 (27.8%)	16/46 (34.8%)	_

RT: radiotherapy; Cardiotoxic chemotherapy: Drug combination that included adriamycin or epirubicin; M: Total mastectomy; O: Ongectomy.

**Table 2:** Comparison of T-SPECT results in breast cancer patients that either received (Yes) or not (No) cardiotoxic adjuvant chemotherapy.

MPI	Left Breast Cancer			Right Breast Cancer			All Breast Cancer		
			
	Yes	No	p	Yes	No	p	Yes	No	p
**SSS**	2.9 ± 3.5	4.7 ± 3.5	0.34	5.2 ± 2.4	3.1 ± 3.1	0.31	3.6 ± 3.3	4.0 ± 3.4	0.83
**SRS**	1.2 ± 1.9	2.0 ± 2.6	0.46	1.2 ± 1.6	0.6 ± 0.6	0.32	1.2 ± 1.7	1.4 ± 2.1	0.97
**ASSS**	1.8 ± 3.6	3.1 ± 3.4	0.45	3.6 ± 0.5	1.1 ± 1.3	0.32	2.4 ± 3.1	2.2 ± 2.8	0.74
**S-RSS**	1.7 ± 1.9	2.6 ± 2.6	0.40	4.0 ± 3.5	2.5 ± 2.7	0.60	2.4 ± 2.6	2.6 ± 2.6	0.79

Cardiotoxic chemotherapy included Adriamycin in 2 patients and Epirubicin in 14 patients; MPI: myocardial perfusion imaging; SSS: summed stress score; SRS: summed rest score; ASSS: anterior wall stress score; S-RSS: the difference of SSS minus the SRS.

Normal MPI scores were observed in 13/28 (46.4%) of left BC patients, 10/18 (55.6%) of right BC patients, and 57/85 (67.1%) of control subjects. Among left BC patients 12/28 (42.9%) had mild, 3/28 (10.7%) had moderate, and none had severe myocardial ischemia on MPI. In the right BC patients 6/18 (33.3%) had mild, 2/18 (11.1%) moderate and none had severe myocardial ischemia. Among the control individuals 28/85 (32.9%) had mild and none had either moderate or severe myocardial ischemia on MPI. The MPI scores in patients with BC in comparison to the control individuals showed increased values of the summed stress score in patients which reached marginal statistical significance only in the left BC patients (Table 3). Comparison of scores between left and right BC patients showed no statistically significant differences but a trend towards increased values of summed rest score in patients with left-sided BC.

**Table 3:** Comparison of myocardial perfusion imaging scores in patients with left/right breast cancer and controls (t-test for independent samples, equal variances not assumed).

MPI	LBC N = 28	RBC N = 18	C N = 85	LBC vs RBC	LBC vs C	RBC vs C
**SSS**	4.0 ± 3.5 (0-13)	3.7 ± 3.0 (0-10)	2.6 ± 2.0 (0-8)	p = 0.78	p = 0.05*	p = 0.14
**SRS**	1.7 ± 2.3 (0-7)	0.8 ± 1.0 (0-4)	1.1 ± 1.3 (0-5)	p = 0.07	p = 0.14	p = 0.40
**ASSS**	2.6 ± 3.5 (0-13)	1.8 ± 1.6 (0-4)	1.7 ± 1.5 (0-6)	p = 0.28	p = 0.18	p = 0.82
**S-RSS**	2.3 ± 2.4 (0-9)	2.9 ± 2.9 (0-9)	1.5 ± 1.6 (0-7)	p = 0.43	p = 0.14	p = 0.06

MPI: myocardial perfusion imaging; LBC: left breast cancer patients; RBC: right breast cancer patients; C: Control individuals; SSS: summed stress score; SRS: summed rest score; ASSS: anterior wall stree score; S-RSS: the difference of SSS minus the SRS; *: p ≤ 0.05.

There were significantly more people in the control group with risk factors for cardiovascular disease such as hypertension, hypercholesterolemia and positive family history of heart disease compared to patient groups (Table 4). After controlling for cardiovascular risk factors, multiple regression analysis demonstrated significant more abnormalities in the SPECT of left BC patients compared to controls in all myocardial perfusion imaging scores evaluated (Table 5). In the right BC patients there were significantly increased myocardial SPECT abnormalities detected by the summed stress score but not with the other perfusion imaging scores. Comparison between left and right BC patients showed no statistical differences between the two groups.

**Table 4:** Cardiovascular risk factors in patients with left and right breast cancer (mean value ± standard deviation).

Risk factor	Left Breast Cancer	Right Breast Cancer	Control individuals	LBC vs Control	RBC vs Control
**Smoking**	2/28 (7.1%)	4/18 (14.3%)	12/85 (14.1%)	p = 0.27	p = 0.46
**HTN**	14/28 (50.0%)	6/18 (21.4%)	54/85 (63.5%)	p = 0.22	p = 0.024*
**DM**	7/28 (25.0%)	4/18 (14.3%)	15/85 (17.6%)	p = 0.51	p = 0.76
**HL**	10/28 (35.7%)	9/18 (32.1%)	49/85 (57.6%)	p = 0.05*	p = 0.57
**Family** **History**	5/28 (17.9%)	5/18 (17.9%)	39/85 (45.9%)	0.003**	0.15

HTN: hypertension; DM: diabetes mellitus; HL: hyperlipidemia; LBC: Left breast cancer; RBC: Right breast cancer; *: p ≤ 0.05; **: p ≤ 0.01

**Table 5:** Multiple regression analysis in 46 female patients with breast cancer and 85 control females after myocardial perfusion imaging with T - SPECT).

Independent variable	SSS Beta	p	SRS Beta	p	ASSS Beta	p
Left BC versus controls	-0.26	0.0001**	-0.02	0.05*	-0.19	0.05*
Right BC versus controls	-0.21	0.045*	0.04	0.68	-0.06	0.59
Left BC versus Right BC	-0.09	0.54	-0.25	0.11	-0.21	0.17

Dependent variables: summed stress score (SSS); summed rest score (SRS); anterior wall stress score (ASSS)

The analysis was performed after controlling for hypertension, hyperlipidemia and family history of cardiovascular disease. BC: breast cancer patients; *: p ≤ 0.05; **: p ≤ 0.01.

## Discussion

Radiation therapy is an integral component in the multidisciplinary management of BC. It has been used for decades to reduce the risk of local-regional recurrence [2]. Adjuvant radiotherapy has also been shown to improve overall survival in patients with advanced-disease stages if the radiation-induced heart disease remains minimal [15]. It is a well known fact that adjuvant chest wall/breast irradiation might be associated with long-term cardiac toxicity, and in the past, a significantly increased rate of non-BC deaths have been reported after RT of left-BC patients [16]. Most of the patients included in these older retrospective analyses were irradiated in 1950s to 1970s, and the increased rate of cardiac mortality was caused by the use of obsolete treatment techniques. By implementation of modern treatment and planning techniques in clinical practice the dose exposure of the heart can be reduced significantly, something anticipated to result in decreased cardiac mortality [17-19]. Thus, it is necessary to protect the heart as much as possible, in order to prevent unnecessary cardiac morbidity [20]. However, breast radiation therapy may still represent a risk factor for cardiovascular disease since some exposure of the heart is unavoidable [21]. Radiation effects to the myocardium are predominantly related to inflammatory changes in the microvasculature, resulting in ischemia, fibrosis and coronary atherosclerosis [22].

In the present study we found significant changes in the myocardial perfusion imaging with T - SPECT of the irradiated BC patients compared to age-matched control individuals. These changes in the multivariate analysis after controlling for the cardiovascular risk factors between cases and controls of hypertension, hyperlipidemia and family history were significantly more pronounced in the left BC patients, where the summed stress score was predominantly reduced followed by the summed rest score and the anterior wall stress score. In addition, the right BC patients were also affected to a lower degree with only the summed stress score showing a marginally significantly reduction compared to controls. However, although all these changes in the left and right BC patients were statistically significant, they were clinically silent and not requiring further action at the time of the evaluation. Even though the clinical consequences of the detected SPECT abnormalities were probably insignificant, further large prospective clinical trials should more accurately assess the toxicity of radiation therapy to BC patients. Our study is in accordance with some prospective studies that examined patients with left BC prior and after radiotherapy. For example, an early prospective study in 12 patients demonstrated myocardial perfusion deficits in 50% of patients 1 year post radiotherapy [23]. Several other more recent prospective studies have reported myocardial perfusion deficits in irradiated left sided BC [24-29]. The findings of some of these studies indicated that the perfusion deficits may appear as early as 6 months post radiotherapy and may involve a large percentage of patients (up to 60%) [24,25]. This is in accordance with our findings that showed no relation between the SSS and time elapsed from radiotherapy to T-SPECT. In addition, these perfusion defects may persist for several years and they may have minimal [27,28], unknown [26], or clinically relevant long-term functional consequences [30]. None of our patients had clinically significant alterations requiring further investigation apart from close observation.

A recently published study reported that for the period 1977-2001 in Denmark the mean heart dose averaged around 6 Gy for left-sided and 2-3 Gy for right-sided radiotherapy and in Sweden decreased from 12.0 to 7.3 Gy for left-sided and from 3.6 to 3.2 Gy for right-sided radiotherapy [31]. Our estimated mean heart dose of 7.7 Gy for left-sided irradiated patients was similar to those observed in the above countries. Other previous studies indicated no cardiotoxicity of radiotherapy in BC patients independently of the site of radiation. Thus, a large retrospective study in 48,353 women with BC, radiotherapy did not increase the risk of myocardial infarction regardless of type of surgery, tumor laterality, or history of cardiac risk factors or heart disease, for at least 10 years follow up [32]. No significant radiation-induced toxicity was also reported in 2 randomized trials of BC patients treated with or without radiotherapy [19,33]. Although superficially it appears that there is discrepancy between the above mentioned studies and our case-control study as well as the other prospective studies that showed myocardial imaging abnormalities induced by radiotherapy [23-29], this is probably not the case since the studies showing no radiotoxicity had as endpoints either myocardial infarction [32] or mortality from ischemic heart disease [19] and most studies showing radiotoxicity as our study concern only abnormal imaging but insignificant clinical consequences.

An important finding in our study is that even clinically silent, there were increased MPI abnormalities in right BC patients after radiotherapy compared to controls. The fact that in our study there was no statistical significance between the left and right irradiated patients suggest that that only comparison between right and left sided irradiated BC patients [34] may not be enough to accurately estimate cardiotoxicity since radiation to either side may result in some MPI changes. Thus, either prospective or case-control studies are probably more reliable to assess the effects of radiotherapy in BC patients.

Apart from possible radiation-induced toxic effects, the heart and coronary vessels may be further compromised in the BC patient by the use of anthracycline-containing chemotherapy in the adjuvant therapy. In the present study, no statistically significant cardiotoxicity was detected in the patients that received adjuvant anthracycline-based chemotherapy compared to patients that did not receive such chemotherapy. In any event, myocardial perfusion scintigraphy, may be an important tool to detect possible myocardial perfusion alterations after radiotherapy [35] or chemotherapy [36] in BC patients.

## Conclusion

The present study demonstrated that postoperative radiation therapy for BC patients result in increased MPI alterations compared to a control group of individuals that had myocardial SPECT for non-specific cardiological complaints. The perfusion alterations although more frequent in the patients that received radiation therapy to the left breast they are also apparent in patients that received radiation to the right breast. However, the clinical consequences of the detected SPECT abnormalities remained unknown and probably insignificant. In any event, asymptomatic BC patients that exhibit a moderately pathologic MPI could be considered for coronary risk factor modification in addition to close observation. Future clinical trials to further assess the toxicity of radiation therapy for BC patients should be prospective or case control studies and not simply comparing toxicity from radiation to left and right side since both therapies may result in some degree of myocardial toxicity.

## Conflicts of interests

The authors declare that they have no competing interests.

### Financial support

None

## Authors' contributions

CS carried out the design of the study and wrote the manuscript. TE, IT, ET, NF, AC worked on the collection and analysis of the data. AF and PT contributed to the conception of the study and the final approval of the final version of the manuscript submitted. AF, PT and TE contributed to the manuscript writing. VR contributed to data interpretation and manuscript writing. All authors read and approved the final manuscript.
